# Interactions Between Two Invertebrate Pathogens: An Endophytic Fungus and an Externally Applied Bacterium

**DOI:** 10.3389/fmicb.2020.522368

**Published:** 2020-11-30

**Authors:** Waqas Wakil, Muhammad Tahir, Abdullah M. Al-Sadi, David Shapiro-Ilan

**Affiliations:** ^1^Department of Entomology, University of Agriculture, Faisalabad, Pakistan; ^2^Department of Entomology, College of Agriculture and Environmental Sciences, The Islamia University, Bahawalpur, Pakistan; ^3^Department of Crop Sciences, College of Agricultural and Marine Sciences, Sultan Qaboos University, Muscat, Oman; ^4^USDA-ARS, SEFTNRL, Byron, GA, United States

**Keywords:** endophytic colonization, *Beauveria bassiana*, *Helicoverpa armigera*, *Bacillus thuringiensis*, chickpea

## Abstract

The members of family Noctuidae exist in diverse environments and many species from this group are of agriculture importance, particularly *Helicoverpa* spp. *Helicoverpa armigera* (Hübner) (Lepidoptera: Noctuidae) is a major pest of many legumes and cereal crops. Due to environmental and regulatory concerns, safe alternatives to broad spectrum chemical insecticides are needed for the control of key noctuid pests such as *H. armigera*. A strain of *Beauveria bassiana* (Cordycipitaceae: Hypocreales) was evaluated for its ability to colonize endophytically in chickpea plants, and its effectiveness against second (L2) and fourth (L4) larval instars of *H. armigera*. *B. bassiana* was inoculated to chickpea plants through injection and endophytic establishment was confirmed by re-isolating the fungi from leaf samples. A detached leaf assay was used to evaluate pathogenicity. *Bacillus thuringiensis* was also applied to both larval stages through leaf dip method. In a novel approach, combined treatments of bacteria and endophytic fungi were compared with single-pathogen treatments. Relative to the single treatments, the combined pathogen treatments exhibited an increase in larval mortality, and decrease in pupation, adult emergence and egg eclosion. Specifically, synergistic effects on mortality were observed when larvae were exposed to simultaneous application of *B. bassiana* (1 × 10^8^ conidia ml^–1^) with *B. thuringiensis* (0.75 μg ml^−1^). Both instars exhibited varying level of growth, development, frass production, diet consumption and fecundity when exposed to the chickpea leaves inoculated with endophytic *B. bassiana* and dipped with sub-lethal doses of *B. thuringiensis*. These findings indicate that the integrated application of endophytic colonized *B. bassiana* and *B. thuringiensis* can be effectively used against *H. armigera*.

## Introduction

Among the leguminous crops chickpea (*Cicer arietinum* L.) is an important, nutritive and inexpensive crop among the people of the developing world (Sharma et al., 2007). Moreover chickpea plays a key role in increasing soil fertility through biological nitrogen fixation (Rao et al., 2008). From sowing to maturity a number of insect pests attack chickpea plants. Among these insect pests *Helicoverpa armigera* (Hübner) (Lepidoptera: Noctuidae) is the most serious (Chaudhary and Chaudhary, 1975; Khan, 1979; Chhabra, 1980). The insect feeds voraciously on tender parts and young pods of the plant and in severe cases can inflict losses up to 100% despite several rounds chemical insecticide applications (Tay et al., 2013). Global economic losses caused by this pest are more than $2 billion annually. To combat this pest, insecticides continue to be the main strategy among the chickpea growers throughout the world. Overuse of chemical insecticides has led to significant resistance issues (Yang et al., 2013). Indeed, low susceptibility toward various chemicals has been reported by a number of scientists (e.g., Cameron et al., 1995; Ahmad et al., 1997; Gunning et al., 1998; Han et al., 1999; Martin et al., 2000). Thus, safe and effective alternative control measures must be sought.

Interest in the use of biopesticides is increasing due to the promising insecticidal properties of these materials and their safety to mammals, birds and other non-target organisms (Inglis et al., 2001). Among biocontrol agents, entomopathogenic fungi are promising measures for safe and long lasting control of voracious pests such as *H. armigera*. Encouraging results of several strains of *Beauveria bassiana* (Balsamo-Crivelli) Vuillemin (Cordycipitaceae: Hypocreales) against different larval stages of lepidopterous insect pests have been recorded (Nguyen et al., 2007). The use of fungi such as *B. bassiana* in different crop plants offers an alternative for the management of *H. armigera* (Sandhu et al., 1993).

An intriguing aspect of entomopathogenic fungi such as *B. bassiana* is that they can exist as endophytes in the plant (Vega et al., 2008). Endophytes are microorganisms that naturally reside within different plant parts (leaves stem and roots) without any apparent disease symptoms (i.e., lesions, retarded growth, and discoloration etc.) (Schulz and Boyle, 2005; Jalgaonwala et al., 2011). *B. bassiana* has been reported worldwide in its distribution, parasitizing various agriculturally important insect pests and has been recovered endophytically from many plants (Vega, 2008). To the benefit of the fungi, endophytically colonized *B. bassiana* receives lifelong protection against environmental factors such as UV light and rain. To the benefit of the plant, a key characteristic of this fungal family (as an endophyte) is the production of secondary metabolites (White et al., 2003), which provide protection against plant pathogens and arthropod pests. Endophytic relationships with entomopathogenic fungi such as *B. bassiana* can be generated by artificially inoculating flowers and or through foliar sprays, rhizome and root immersion, soil drenching, radical dressing and seed dressing (Parsa et al., 2013).

The efficacy of endophytic colonization by *B. bassiana* for control of various insect pests in different field crops has gained recent attention (Qayyum et al., 2015). The novel approach to introducing entomopathogenic fungi into a system demonstrates a variety of symbiotic relationships with host plants, and exploits these interactions to open new perspectives regarding pest management and improving plant health. Numerous studies have confirmed the protection by endophytic fungi against plant diseases, insects (Arnold et al., 2003; O’Hanlon et al., 2012) or plant parasitic nematodes (Waweru et al., 2014). A number of success stories of endophytic relationships in banana (Akello et al., 2008), date palm (Gómez-Vidal et al., 2006), cacao (Posada and Vega, 2005), coffee (Posada et al., 2007), jute (Biswas et al., 2012), maize (Cherry et al., 2004), pecan (Ramakuwela et al., 2019), potato (Jones, 1994), sorghum (Tefera and Vidal, 2009), tomato (Leckie, 2002; Qayyum et al., 2015) and *Theobroma gileri*, a close relative of cocoa (Evans et al., 2003) has confirmed broad utility of the approach in controlling insect pests of many important crops. The symbiotic relation of the fungal entomopathogen *B. bassiana* has become an integral part of *Conopomorpha cramerella* (Snellen) (Lepidoptera: Gracillariidae) management in Indonesia, Malaysia and Philippines (Posada and Vega, 2005). The introduction of *B. bassiana* as fungal endophyte in maize had success in providing control of *Ostrinia nubilalis* (Hübner) (Arnold and Lewis, 2005). Endophytic *B. bassiana* has been shown to induce infection and protect against *H. armigera* in prior studies though not in a chickpea system (Qayyum et al., 2015).

Another promising group of biological control agents for noctuid pests such as *H. armigera* are entomopathogenic bacteria from the genus *Bacillus*. These bacteria are key antagonists of numerous insect pests of economic importance in various cropping systems (Salama et al., 2004). *Bacillus thuringiensis* is often an integral part of products used in biological control strategies worldwide; about 90% microbial pesticides used globally are *B. thuringiensis* with annual sales of about $3 billion (although that is only 5% of total crop protection market) (Marrone, 2014; Kumar and Singh, 2015; Olson, 2015; Damalas and Koutroubas, 2018). Numerous species of this genus particularly *B. thuringiensis* Berliner (Bt) are frequently used against a vast array of insect pests from the orders Coleoptera, Lepidoptera and Diptera etc; these bacteria exhibit a high degree of specificity toward the host and specific stage of the host (Salama et al., 2004).

Combined application of more than one biocontrol agent can result in synergistic levels of pest control. For example, Wraight and Ramos (2005) observed that integrated application of *B. bassiana* and *B. thuringiensis* resulted in synergistic interactions against some insect species. Later, in another study, synergistic interactions between *B. bassiana* and *B. thuringiensis* were confirmed against Colorado potato beetle (*Leptinotarsa decemlineata* Say), which was consistent under varying environmental regimes (Wraight and Ramos, 2017). Similar results were reported from the combined application of both agents against Mediterranean flour moth larvae (Sandner and Cichy, 1967). However, there is a dearth of information on using *B. thuringiensis* in combination with entomopathogenic fungi in their endophytic state. The present study targeted *H. armigera* infestation in chickpea managed through endophytic colonization of *B. bassiana* alone and in combination with *B. thuringiensis*, impact of treatments on the development, diet consumption, frass production and weight gain was also assessed.

## Materials and Methods

### *Helicoverpa armigera* Collection and Rearing

Larvae of *H. armigera* for the experiments were obtained from the culture collection of the Microbial Control Laboratory, Department of Entomology, University of Agriculture, Faisalabad, Pakistan. The culture was maintained in the laboratory for more than 30 generations on an artificial diet (Wakil et al., 2011). The diet consists of chickpea flour (125 g), red kidney beans (125 g), canned tomato paste (25 g), yeast (40 g), agar (17 g), and a vitamin mixture (10 ml) mixed thoroughly in distilled water (1300 ml). Due to their cannibalistic nature larvae were reared individually in plastic vials (7 cm height, 3 cm in diameter). After pupation, they were transferred to small plastic boxes for adult emergence. The adult’s diet contained 10% honey solution. The rearing conditions were maintained at 25°C and 65% RH in an incubator (MIR-254, Sanyo, Japan).

### Isolation of Endophytic Fungi

For isolation of endophytic fungi, chickpea fields were visited in different chickpea growing areas of Punjab, Pakistan. The plants were selected on the basis of cadavers lying under the plants indicating the potential presence of endophytic fungi. Endophytically colonized fungi were isolated following the method of Arnold et al. (2000). Before isolation, plants were carefully uprooted, sealed in a zipper bag, transferred to the laboratory and stored at 4°C until processing. For isolation, leaves were first washed with running tap water and dried for 10 min. A small piece (3 mm^2^) was cut from the center of the leaf with sterilized scissors followed by surface sterilization with 70% ethanol for 2 min followed by 2% sodium hypochlorite for 3 min and then 3 rinses in distilled water. Leaf pieces were dried on sterilized tissue paper and placed on Petri dishes containing 20 ml of Potato Dextrose Agar (PDA) (Merck, Germany) amended with chloramphenicol (50 μg ml^–1^), streptomycin sulfate (50 μg ml^–1^) (Sigma, St Louis, MO, United States) and 0.5 g L^–1^ of dodine (65%). After 14 days on the media plates, leaf pieces that exhibited fungal growth were transferred to glass tubes on Sabouraud Dextrose Agar (SDA) (BD, France) media slants for storage and future use. The fungus was identified (Barnett and Hunter, 1999) as *B. bassiana* on morphological characteristics: conidia were produced in clusters with a characteristic snowball shape and the conidiogenous cells had a flask shaped basal section with a zig zag rachis.

### Conidial Suspension

*Beauveria bassiana* isolate was mass cultured in PDA medium enriched with 1% yeast extract (Watson et al., 1995) containing 50 μg ml^–1^ of chloramphenicol. The plates were incubated at room temperature and humidity level (25°C and 65% RH), 14 h Light: 10 h Dark photoperiod. Conidia from each plate were harvested by scraping with a sterilized scalpel, and conidial powder was dried and stored at 4°C until formulation and use. For experimentation, conidial powder was suspended in sterile distilled water containing 0.01% (v/v) Tween-80 (Merck, Germany). The conidia were dislodged into the suspension with a glass rod. The suspension was filtered through a double layer of sterile cheesecloth (mesh: 36 by 36 threads cm^–2^) to remove clumps of mycelia. The conidial suspension was poured into a sterile glass tube and vortexed for spore viability determination (Lezama-Gutiérrez et al., 2001). Spore concentration was determined with a hemocytometer and serial conidial dilutions were made until required conidial concentrations were achieved. The germination rate of *B. bassiana* conidia was >93% determined prior to each bioassay.

### Colonization of Endophytic Fungi in Chickpea Plants

The *B. bassiana* isolate was inoculated in chickpea seedlings via injection of a conidial suspension into the plant. The chickpeas were grown in a glass house at the University of Agriculture, Faisalabad. Two spore concentrations (1 × 10^5^ and 1 × 10^8^ conidia ml^–1^) for colonization were prepared in 0.01% Tween-80. Fifteen plants were selected for each replication and three replications were used for each treatment. Forty-day old chickpea plants were selected for injection; injections were made to the stem base with 1 ml of respective doses of fungal suspension using a sterile syringe. Control plants were injected with 1 ml of sterile water containing 0.01% Tween-80. The entire experiment was repeated three times.

### Preparation of *B. thuringiensis* Spore-crystal Mixtures

The strain of *B. thuringiensis* isolate was obtained from the Microbial Control Laboratory collection (Department of Entomology, University of Agriculture, Faisalabad) and was originally obtained from National Center for Genetic Engineering and Biotechnology (BIOTEC) in Thailand. This strain was then subjected to sporulation by culturing in 50 ml nutrient broth media. Harvesting of cultures was carried out by centrifugation at 6000 rpm for 15 min (Crecchio and Stotzky, 2001; Hernández et al., 2005). The pellets received three washings with cold 1 M NaCl and re-suspended. Spore concentration was adjusted by 1:100 dilutions and optical density was measured at 600 nm (Hernández et al., 2005), then the samples were stored in refrigerator until used (Wakil et al., 2013).

### Mortality Assays

Bioassays were conducted to assess the endophytic efficacy of *B. bassiana* against second (L2) and fourth (L4) instar larvae of *H. armigera*. Fifteen leaves from inoculated (three weeks earlier) and un-inoculated (control) plants were detached and offered to L2 and L4 instar *H. armigera* larvae. For *B. thuringiensis* treatments, three concentrations (0.50, 0.75, and 1.0 μg ml^–1^) were prepared. A piece of chickpea leaf (2 × 2 cm^2^) was immersed in respective doses of *B. thuringiensis* suspension for 60s and offered to the respective larval stages individually. The larvae were allowed to feed on treated leaves for 48 h and shifted to the artificial diet for rest of the period. In combined treatments *B. bassiana* inoculated leaves were immersed in respective doses of *B. thuringiensis* suspension for 60 s and then offered to the larvae. Mortality was recorded after every 48 h up to pupation for both larval instars. The larvae were prodded with a blunt needle and those unable to move in a coordinated manner were considered as dead (Ma et al., 2008). From the surviving individuals, percentage pupation, adult emergence and egg eclosion were also recorded. Three replicates of fifteen larvae were used for each treatment and same count of larvae fed on normal leaves served as the non-treated check. All the treatments were incubated at 25°C and 65% RH and 14 h Light: 10 h Dark photoperiod in an incubator (Sanyo, Japan). The entire experiment was repeated three times.

### Effect of Sub-lethal Doses on Development of *H. armigera*

To determine the effect of sub-lethal doses of endophytic *B. bassiana* (1 × 10^5^ conidia ml^–1^) and *B. thuringiensis* (0.20, 0.30, and 0.40 μg ml^–1^) on developmental parameters viz. larval duration, larval weight, pre-pupal duration, pre-pupal weight, adult longevity (male and female) and adult weight (male and female) was recorded on L4 instar larvae of *H. armigera*. The sub-lethal doses were selected based on preliminary assays. A piece of endophytically colonized *B. bassiana* and *B. thuringiensis* were applied alone and in combination against L4 instar larvae and incubated at above mentioned conditions. The larvae were allowed to feed on treated leaves for 48 h and then shifted to the artificial diet for rest of the period. After pupation, the pupae were transferred to separate small plastic jars for adult emergence. The adults were fed on 10% honey solution. Adults were observed on daily basis and mortality was determined until the death of last adult. Each treatment consisted of 45 larvae with three replicates and the experiment was repeated thrice. The environmental conditions were set at 25°C and 65% RH and 14 h Light: 10 h Dark photoperiod inside the incubator.

### Effect of Sub-lethal Doses on Diet Consumption, Weight Grain and Frass Production

A new batch of 6th instar *H. armigera* (L6) were offered sub-lethal doses of endophytic *B. bassiana* (1 × 10^5^ ml^–1^) and *B. thuringiensis* (0.20 μg ml^–1^). A piece of endophytically colonized chickpea leaf alone and in combination with *B. thuringiensis* was offered to the larvae. The larvae were allowed to feed on treated leaves for 48 h and then shifted to the artificial diet for rest of the period. Before exposing to the leaves each larva was weighed and transferred to a rearing vial. Every day until the larvae pupated or died, larvae were moved to new vials individually and provided with fresh diet every day. Frass produced during this period was separated from vials using the tip of a fine camel hair brush; the frass was then weighed. Diet left unused in each vial was recovered and dried in a drying oven at 80°C. Prior to the assay, diet in 30 cups was dried to obtain an estimate of the original dry weight. Diet consumption of each larva was determined by subtracting the weight after feeding from before. Moreover, total frass production and weight gain during this period were also determined.

### Statistical Analysis

Percentage mortality was recorded and corrected for control mortality using Abbott (1925) formula. The data were then subjected to a one-way analysis of variance in Minitab statistical package (Minitab 2003) using Tukey’s Kramer test (HSD) (Sokal and Rohlf, 1995) at 5% significance level. The type of interaction between combined treatments of *B. thuringiensis* and *B. bassiana* was determined by CTF analysis; CTF = (Oc-Oe)/Oe × 100, where CTF is the co-toxicity factor, Oc is the observed mortality (%) in combined application, and Oe the expected mortality (%), that is the sum of individual mortality (%) encountered in each of the treatments used in the combination (Mansour et al., 1966). The interactions were categorized as additive, synergistic or antagonistic: CTF ≥ 20 indicates synergism, CTF > −20 indicates additivity, and CTF < −20 indicates antagonism (Mansour et al., 1966; Wakil et al., 2012).

## Results

### Mortality Assays

Treatment effects were detected in the mortality assays. Table 1 presents the statistical results from the factorial analysis including treatment and interaction effects. Table 2 presents mean mortalities and the multiple range distribution (HSD test). Significant differences were recorded for mortality among different treatments and instars (treatment: *F*_7_, _160_ = 199.67, *P* ≤ 0.05; instar: *F*_1_, _161_ = 64.21, *P* ≤ 0.05) but a non-significant interaction was recorded for instar × treatment (*F*_7_, _161_ = 1.54, *P* = 0.09) (Table 1). A synergistic effect (CTF ≥ 20) on mortality was observed when larvae were exposed to simultaneous application of endophytic *B. bassiana* and 0.75 μg ml^–1^ of *B. thuringiensis* in case of both instars tested (Table 2).

**TABLE 1:** Factorial analysis of mortality, pupation, adult emergence and egg eclosion of *H. armigera* treated with endophytically colonized *B. bassiana* and *B. thuringiensis.*

Source	*df*	Mortality		Pupation		Adult emergence		Egg eclosion	
					
		*F*	*P*	*F*	*P*	*F*	*P*	*F*	*P*
Instar	1	64.21	≤0.05	61.37	≤0.05	54.23	≤0.05	36.21	≤0.05
Treatment	7	199.67	≤0.05	222.34	≤0.05	213.29	≤0.05	249.0	≤0.05
Instar × treatment	7	1.54	0.09	0.58	0.53	0.59	0.67	0.67	0.38
Error	145	-	-	-	-	-	-	-	-
Total	160	-	-	-	-	-	-	-	-

**TABLE 2:** Mortality (mean ± SE)% of L2 and L4 instar *H. armigera* treated with endophytic *B. bassiana* (Bb: 1 × 10^8^ conidia ml^−1^) alone and in combination with *B. thuringiensis* (Bt1: 0.50; Bt2: 0.75; Bt3: 1.0 μg ml^−1^).

Treatments	Second instar				Fourth instar			
		
	Observed mortality (%)	Expected mortality	CTF	Type of interaction	Observed mortality (%)	Expected mortality	CTF	Type of interaction
Bb	27.13 ± 1.36d				19.69 ± 1.19e			
Bt1	19.96 ± 1.11d				14.87 ± 1.07e			
Bt2	33.64 ± 2.02d				25.74 ± 1.25de			
Bt3	58.05 ± 2.80bc				49.36 ± 2.34bc			
Bb + Bt1	49.07 ± 2.13c	47.09	4.03	Additive	35.92 ± 1.70cd	34.56	3.78	Additive
Bb + Bt2	77.40 ± 3.10a	60.77	21.48	Synergistic	58.82 ± 2.77a	45.43	22.76	Synergistic
Bb + Bt3	68.38 ± 3.03ab	85.18	−24.5	Antagonistic	53.45 ± 2.31ab	65.05	−21.70	Antagonistic
*df*	6		-	-	6		-	-
*F*	40.6		-	-	31.6		-	-
*P*	≤ 0.05		-	-	≤0.05		-	-

*Means followed by the same letters within each column are not significantly different; HSD test *P* ≤ 0.05.*

There were higher numbers of dead larvae observed when L2 instar exposed to the treatments than L4 instar larvae (Table 2). The combined application of endophytic *B. bassiana* and *B. thuringiensis* proved more fatal to both instars compared to single application of each agent. For instance, the highest mortality (77.40%) for L2 instar larvae was observed where simultaneous application of *B. bassiana* and *B. thuringiensis* (0.75 μg ml^–1^) was applied followed by 68.38% when treated with *B. bassiana* and *B. thuringiensis* (1.0 μg ml^–1^) and 49.07% when treated with *B. bassiana* (1 × 10^8^ conidia ml^–1^) and *B. thuringiensis* (0.50 μg ml^–1^) (Table 2). Additive effects (CTF ≤ 20) were recorded when the tested instars were treated with low doses of *B. thuringiensis* in integration with endophytic *B. bassiana* while an antagonistic effect was observed at highest dose of *B. thuringiensis* and endophytic *B. bassiana* (Table 2). A similar trend in mortality was recorded for both 2nd and 4th instars.

**FIGURE 1:**
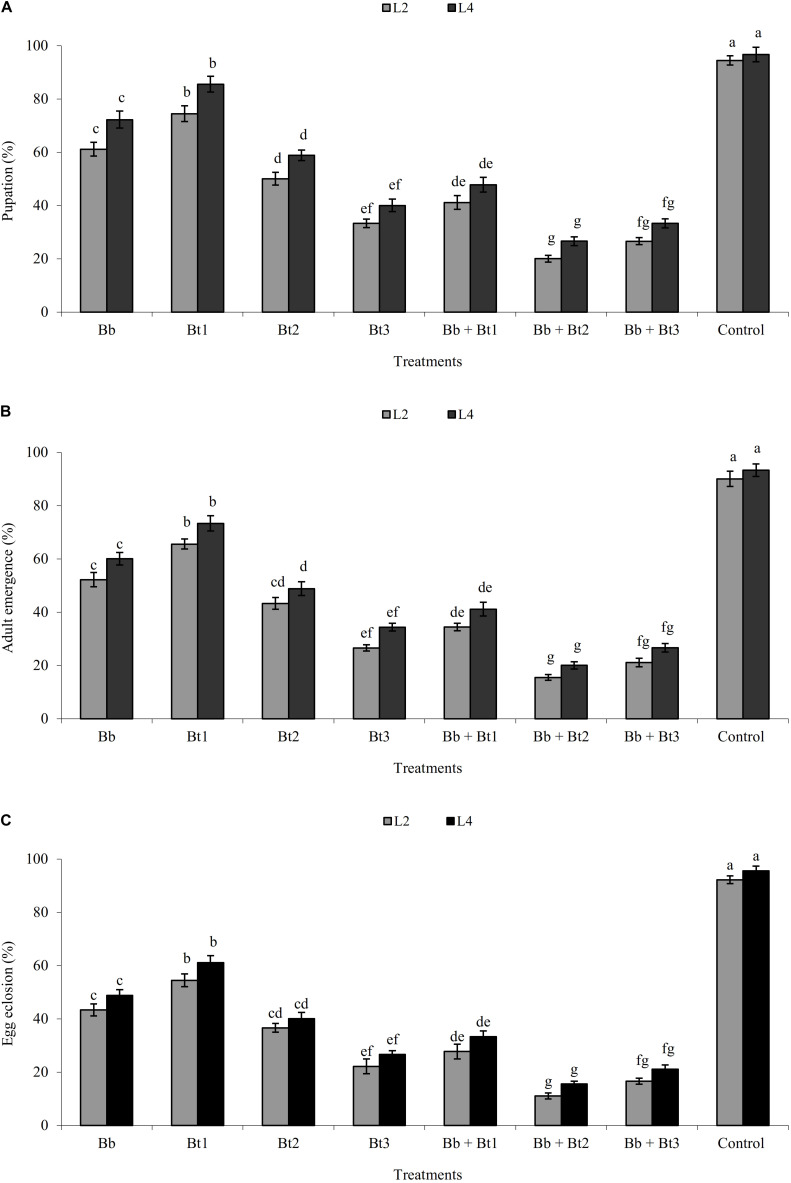
Pupation **(A)**, adult emergence **(B)**, and egg eclosion **(C)** of L2 and L4 instars (mean ± SE)% of *H. armigera* treated with endophytic *B. bassiana* (Bb: 1 × 10^8^ conidia ml^–1^) alone and in combination with *B. thuringiensis* (Bt1: 0.50; Bt2: 0.75; Bt3: 1.0 μg ml^–1^). Means followed by the same letter within each larval instar are not significantly different; HSD test *P* ≤ 0.05.

Percentage pupation, adult emergence and egg eclosion from surviving individuals generally decreased within single and combined treatments as the dose rate of *B. thuringiensis* increased (Figure 1). Overall, pupation, adult emergence and egg eclosion was lower in the combination treatments than the single-applied treatments (Figure 1).

### Effect of Sub-lethal Doses on Development of *H. armigera*

When larvae were encountered different sub-lethal concentrations of *B. thuringiensis* and *B. bassiana* (1 × 10^5^ conidia ml^–1^) and endophytically colonized chickpea leaves, significant variation was recorded for all factors including incubation of egg period: *F*_7_, _71_ = 45.4, *P* ≤ 0.05; larval duration: *F*_7_, _71_ = 17.3, *P* ≤ 0.05; pre-pupal duration: *F*_7_, _71_ = 30.70, *P* ≤ 0.05; pupal duration: *F*_7_, _71_ = 21.90, *P* ≤ 0.05; total immature period: *F*_7_, _71_ = 63.1, *P* ≤ 0.05; pre-oviposition period: *F*_7_, _71_ = 10.8, *P* ≤ 0.05; oviposition period: *F*_7_, _71_ = 36.4, *P* ≤ 0.05; post-oviposition period: *F*_7_, _71_ = 16.0, *P* ≤ 0.05; daily fecundity: *F*_7_, _71_ = 214.0, *P* ≤ 0.05; total fecundity l: *F*_7_, _71_ = 1902.0, *P* ≤ 0.05; adult longevity: (female: *F*_7_, _71_ = 30.3, *P* ≤ 0.05 and male: *F*_7_, _71_ = 35.0, *P* ≤ 0.05). Overall, incubation of eggs, larval, pre-pupal period, pupal duration, total immature period and pre-oviposition period increased as the dose rate of *B. thuringiensis* application increased, and these parameters tended to be shorter for the combine treatments than the individual treatments (Table 3). In contrast, oviposition, post-oviposition period, fecundity daily, fecundity total and adult longevity (male and female) decreased as the dose rate of *B. thuringiensis* application increased. Here again the combined treatments were more effective than single treatments (Table 3).

**TABLE 3:** Eggs incubation (days), larval duration (days), pre-pupal duration (days), pupal duration (days), total immature period (days), oviposition period (days), fecundity (eggs), and adult longevity (days) (mean ± SE)% of L2 instar *H. armigera* treated with endophytic *B. bassiana* (Bb: 1 × 10^5^ conidia ml^–1^) alone and in combination with *B. thuringiensis* (Bt1: 0.20; Bt2: 0.30; Bt3: 0.40 μg ml^–1^).

Developmental period	Bb	Bt1	Bt2	Bt3	Bb + Bt1	Bb + Bt2	Bb + Bt3	Control
Incubation of eggs (days)	1.820 ± 19def	1.700 ± 15ef	1.960 ± 09cde	2.270 ± 06abc	2.120 ± 07bcd	2.560 ± 08a	2.420 ± 05ab	1.610 ± 09f
Larval duration (days)	18.210 ± 94de	17.881 ± 03de	19.000 ± 55cde	20.770 ± 46bc	19.440 ± 58cd	23.440 ± 37a	22.440 ± 53ab	17.000 ± 62e
Pre-pupal duration (days)	4.240 ± 27def	4.110 ± 54ef	4.380 ± 09cde	4.530 ± 07cd	4.670 ± 03bc	5.120 ± 04a	4.970 ± 06ab	3.920 ± 09f
Pupal duration (days)	12.641 ± 05def	11.841 ± 02ef	13.330 ± 37cde	14.770 ± 27abc	14.000 ± 23bcd	15.770 ± 27a	15.200 ± 22ab	11.550 ± 37ef
Total immature period (days)	36.851 ± 42de	35.571 ± 55ef	38.660 ± 33cd	42.330 ± 57b	40.220 ± 46bc	47.000 ± 86a	45.000 ± 57a	34.110 ± 63f
Pre-oviposition period (days)	3.050 ± 26cde	3.040 ± 27de	3.130 ± 07cde	3.320 ± 07abc	3.230 ± 05bcd	3.540 ± 06a	3.430 ± 05ab	2.920 ± 06e
Oviposition period (days)	12.461 ± 37ab	11.881 ± 15ab	11.000 ± 28bc	9.110 ± 26de	10.110 ± 26cd	7.330 ± 23f	8.220 ± 36ef	13.000 ± 50a
Post-oviposition (days)	0.920 ± 09bcd	1.020 ± 11abc	0.840 ± 03cde	0.680 ± 04e	0.770 ± 04de	1.150 ± 02a	1.030 ± 04ab	1.100 ± 03ab
Fecundity-daily (eggs)	51.352 ± 40b	53.112 ± 45b	48.250 ± 32c	42.880 ± 51d	46.330 ± 44c	37.550 ± 37f	40.110 ± 53e	55.660 ± 40a
Fecundity-total (eggs)	826.2648 ± 32b	840.5652 ± 21b	792.2143 ± 52c	661.7848 ± 14e	737.6750 ± 20d	456.4441 ± 14g	591.7845 ± 69f	861.1152 ± 31a
Adult longevity (days)								
Male	18.121 ± 12ab	17.880 ± 30ab	16.110 ± 35cd	15.000 ± 33de	16.660 ± 33bc	13.220 ± 27f	13.660 ± 33ef	19.330 ± 52a
Female	16.471 ± 24a	15.770 ± 40ab	14.880 ± 26bc	13.220 ± 22de	14.110 ± 26cd	12.000 ± 28e	12.770 ± 27de	17.1110 ± 42a

*Means followed by the same letter within each column are not significantly different; HSD test *P* ≤ 0.05.*

### Effect of Sub-lethal Doses on Diet Consumption, Weight Grain and Frass Production

**FIGURE 2:**
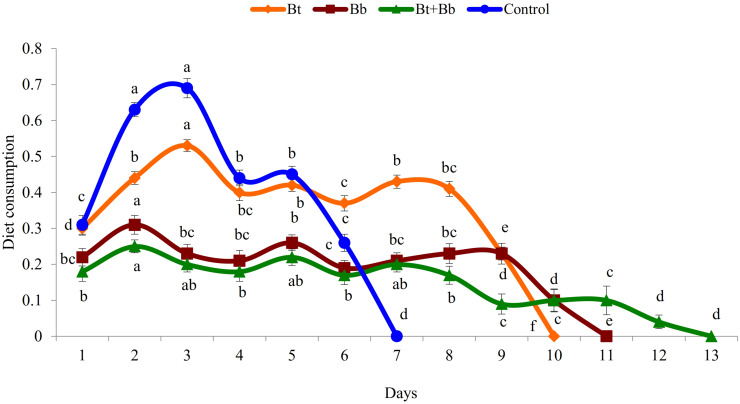
Diet consumption (g) in last instar *H. armigera* treated with endophytic *B. bassiana* (Bb: 1 × 10^5^ conidia ml^–1^) alone and in combination with *B. thuringiensis* (Bt1: 0.20 μg ml^–1^). Means followed by the same letter within each treatment are not significantly different; HSD test *P* ≤ 0.05.

**FIGURE 3:**
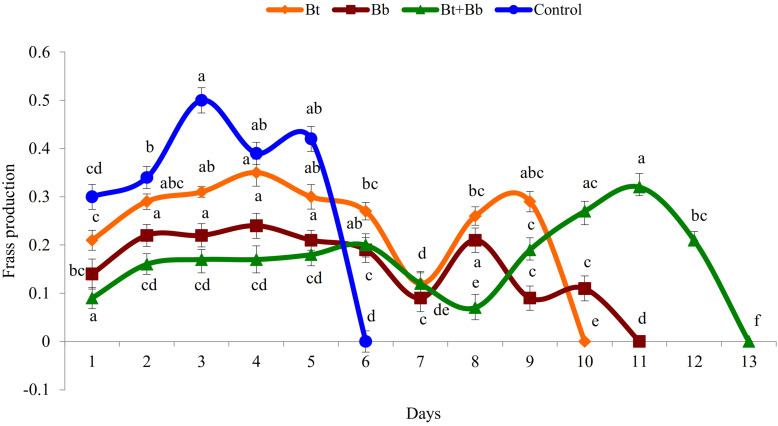
Frass production (g) in last instar *H. armigera* treated with endophytic *B. bassiana* (Bb: 1 × 10^5^ conidia ml^–1^) alone and in combination with *B. thuringiensis* (Bt1: 0.20 μg ml^–1^). Means followed by the same letter within each treatment are not significantly different; HSD test *P* ≤ 0.05.

**FIGURE 4:**
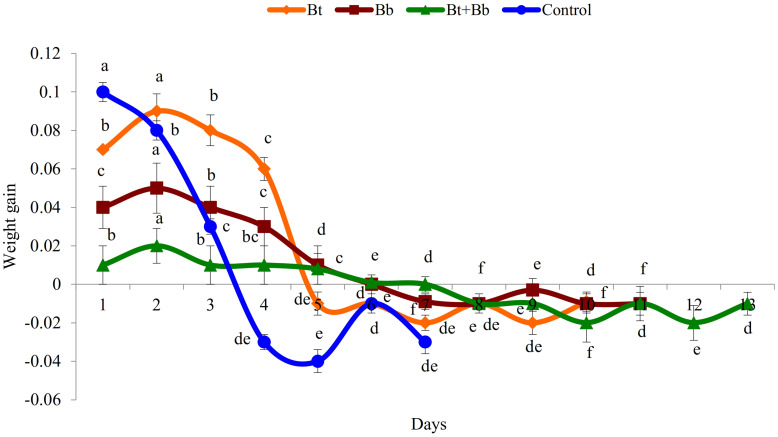
Weight gain (g) in last instar *H. armigera* treated with endophytic *B. bassiana* (Bb: 1 × 10^5^ conidia ml^–1^) alone and in combination with *B. thuringiensis* (Bt1: 0.20 μg ml^–1^). Means followed by the same letter within each treatment are not significantly different; HSD test *P* ≤ 0.05.

Diet consumption by 6th instar larvae was significantly influenced by the treatments (Bt: *F*_9_, _89_ = 169.0, *P* ≤ 0.05; Bb: *F*_10_, _98_ = 56.5, *P* ≤ 0.05; Bt + Bb: *F*_12_, _116_ = 46.8, *P* ≤ 0.05; Control: *F*_6_, _62_ = 167.0, *P* ≤ 0.05); diet consumption was lower in combined treatments compared with sole applications. Diet consumption was the lowest for the endophytic *B. bassiana* and *B. thuringiensis* while the highest consumption was recorded in the control treatment (Figure 2). Frass production was also significantly influenced by treatments applied (Bt: *F*_9_, _89_ = 39.4, *P* ≤ 0.05; Bb: *F*_10_, _98_ = 44.5, *P* ≤ 0.05; Bt + Bb: *F*_12_, _116_ = 44.7, *P* ≤ 0.05; Control: *F*_6_, _62_ = 178.0, *P* ≤ 0.05). More frass production was recorded during the initial days of treatments and gradually decreased to zero before pupation. On the other hand, the highest level of frass production was found in non-treated larvae (Figure 3). Larvae treated with sub-lethal single-treatment concentrations of *B. bassiana* and *B. thuringiensis* gained more weight compared to their combined application (Figure 4). Weight gain was also significantly different among the treatments (Bt: *F*_9_, _89_ = 734.0, *P* ≤ 0.05; Bb: *F*_10_, _98_ = 529.0, *P* ≤ 0.05; Bt + Bb: *F*_12_, _116_ = 166.0, *P* ≤ 0.05; Control: *F*_6_, _62_ = 175.0, *P* ≤ 0.05).

## Discussion

Our study indicated that *B. bassiana* successfully colonized chickpea plants endophytically and caused considerable mortality in *H. armigera* larvae. Similarly, successful colonization of *B. bassiana* in corn plant tissue resulted in high mortality in larvae of *O. nublialis* when encountering endophytically colonized plants (Bing and Lewis, 1991, 1992a,b). In banana plants endophytically colonized *B. bassiana* caused high larval mortality in banana root borer; eggs and adults that emerged from infected larvae showed mycosis (Akello et al., 2008). In another example, mycosed larvae of *H. zea* were also reported from tomato plants inoculated with *B. bassiana* (Powell et al., 2009). The authors (Powell et al., 2009) also observed fungal infection within *H. armigera* larvae and later confirmed this by visual fungal growth on cadavers after feeding on tomato (Powell et al., 2007). The injection of *B. bassiana* and subsequent pathogenic endophyte status against the target pest, *H. armigera*, has been successfully demonstrated previously (Qayyum et al., 2015). In the current study we are not necessarily advocating injection as a practical means where a farmer would go out and infect every single plant in his or her field. Rather, we are putting forth this study as a model system indicating the unique synergistic interaction between *B. thuringiensis* and *B. bassiana* when it is in the plant as an endophyte.

Endophytic entomopathogenic fungi mostly belong to order Hypocreales of division Ascomycota (Roy et al., 2006). They are classified as Class II endophytes due to the ability to endophytically colonize both above and below the ground tissue of the host plant (Rodriguez et al., 2009; Quesada-Moraga et al., 2014). Entry into the plant stems from the germ tube formed from conidia, from which fungal hyphae produce and penetrate into the plant tissues through the stomata or with the aid of fungal enzymes and turgor pressure produced from the hyphae via the epidermal cells (Wagner and Lewis, 2000). The main objective of our study was to evaluate the effectiveness of *B. bassiana* endophytically colonized into chickpea plants against *H. armigera* and therefore we did not investigate the factors influencing fungal penetration and its colonization inside the plants. However, literature reveals that researchers have investigated different plant/leaf factors affecting the colonization of endophytic fungi. Endophytic fungi have been reported in every plant species examined to date (Tejesvi et al., 2007) and colonize vegetative and reproductive parts of their host plants (Arnold et al., 2003). Cannon and Simmons (2002) observed that endophytic colonization of species of *Colletotrichum*, *Nodulisporium*, *Pestalotiopsis*, and *Phomopsis* was greater in the midrib than in laminar tissue, and slightly greater at the tip of the lamina compared with the base of the leaves. Some other studies have been conducted to evaluate the richness and the distribution of endophytes in the plant leaves and found that some taxa were leaf age specific and composition of endophytes varied with leaf region (Hilarino et al., 2011). Other studies indicate that old leaves support more endophytes than younger leaves (Suryanarayanan and Thennarasan, 2004). Fernandes et al. (2011) reported that changes in leaf biochemistry influenced endophytic colonization with consequences for endophyte distribution. On the other hand, Arnold and Herre (2003) argued that leaf chemistry has a minor role in endophyte colonization. The relation of a polyphenolic biomolecule “tannin” with endophytic fungal colonization in the leaves of *Bauhinia brevipes* (Fabaceae) has also been studied (Cornelissen and Fernandes, 2001). It would indeed be interesting to know what other fungi colonize the plant along with *B. bassiana*, which is an interesting point for further study.

The mode of action of endophytic entomopathogenic fungi is still unclear but most of the researchers believe that the endophytic entomopathogenic fungi do not cause direct infection rather mortality results from feeding deterrence or antibiosis through inducing systemic plant defenses. There are many studies involving the artificial colonization of endophytic fungi in different plants and subsequent mortality of target insect species. For example, two strains of *B. bassiana* were successfully established as endophytes in *Citrus limon* (L.) Osbeck plants as indicated through systemic colonization of the various citrus plant parts i.e., leaves, stems and roots by BB Fafu-13 strain. Endophytic *B. bassiana* induced 10% to 15% mortality within 7 days of exposure when adult psyllids that were allowed to feed on the leaves of treated plants, and there was no mycosis detected on any of the dead psyllids (Bamisile et al., 2019). In another study, very low levels of mycosis (5.4–9.2%) was detected from cadavers of horse-chestnut leaf miner *Cameraria ohridella* Deschka & Dimić from two different strains of *B. bassiana* AM-EF0111 and AM-EP0715 (Barta, 2018). Jaber et al. (2018) reported no fungal outgrowth in sweetpotato whitefly *Bemisia tabaci* Gennadius when exposed to endophytically colonized *B. bassiana* and *Metarhizium brunneum*. Similar findings have already been reported by other researchers where no fungal outgrowth was discovered in cadavers of insects fed with endophytically colonized plants (Resquín-Romero et al., 2016; Sánchez-Rodríguez et al., 2018). Many other studies showed no mycosis or only rare instances of mycosis from the cadavers (Cherry et al., 2004; Quesada-Moraga et al., 2009; Resquín-Romero et al., 2016; Sánchez-Rodríguez et al., 2017). In our study we also did not observe fungal outgrowth in *H. armigera* colonized with *B. bassiana*; therefore, we hypothesize that toxins produced by *B. bassiana* are responsible for the mortality of test insect species. *Beauveria bassiana* produces beauvericins and bassianolide, beauveriolides, oosporein, tenellin, pyridovericin, pyridomacrolidin, bassiacridin and bassianin. By forming complexes with cations, beauvericin causes an increase in permeability of natural and artificial membranes. Beauvericin also induces programmed cell death similar to apoptosis (Waetjen et al., 2014). The toxin was cytotoxic (IC50 0.5 μM) to a *Spodoptera frugiperda* (SF-9) cell line (Fornelli et al., 2004) and on the Colorado potato beetle (LC_50_ 633 ppm) (Roberts et al., 1992). Feng et al. (2015) verified the role of oosporein in fungal virulence to host insects through the inhibition of insect defense mechanisms.

*Bacillus thuringiensis* toxins have also been found to be lethal to a vast array of insect pests belonging to the orders Coleoptera, Diptera, and Lepidoptera (Feitelson et al., 1992; Schnepf et al., 1998), and hence *B. thuringiensis*-spore crystal mixtures have been in used widely as bio-pesticides. For infection to occur *B. thuringiensis* toxins attach to the specific bindings sites of the insect’s midgut which then leads to cell lysis. This lysis may result in the insect’s cessation of feeding, lethargy and ultimately death (Marzban et al., 2009). The integrated use of entomopathogenic fungi and Bt has depicted effective control of insect pests (Mwamburi et al., 2009; Wakil et al., 2013), however, no comparable literature is available regarding the combination of endophytic fungi with *B. thuringiensis* against any insect species.

Integration of two or more entomopathogens to control insect pests may increase efficacy and the chances of targeting multiple hosts (Pingel and Lewis, 1999). Marzban et al. (2009) reported that the integration of two or more myco-pathogens interact positively relative to their individual effect. In many combinations, the virulence of an agent is enhanced by the action of the other agent, which resultantly increases the speed or magnitude of kill, and causes retarded growth and reduced feeding in the host. In combined treatments of *B. thuringiensis* and *B. bassiana*, both agents work synergistically weakening the insect and affecting the insect immune response to allow entomopathogens to infect the host more efficiently. When *B. bassiana* gains access to the insect gut, it boosts the infection of *B. thuringiensis* toxins. In this way both agents help each other in retardation of normal physiological functions of an insect host. These findings are further supported by Allee et al. (1990) who reported that *B. bassiana* germinating and invading the insect favors the activity of *B. thuringiensis* toxins to increase pathogenic severity in grubs of Colorado potato beetle.

The findings of our study revealed higher mortality levels of *H. armigera* larvae in combined treatments of *B. bassiana* inoculated chickpea leaves and *B. thuringiensis* compared to their sole applications. Synergistic interactions were recorded at intermediate doses of *B. thuringiensis* while, additive at low doses and antagonistic effects were observed at high dose rates. It would be interesting to explore why antagonism was observed at the highest dose that investigation is beyond the scope of this current study. Thus, as observed previously, interactions among entomopathogens can vary depending on the dosage of the microbial agents applied (Shapiro-Ilan et al., 2004). Synergistic interactions were reported by Wraight and Ramos (2005) when *B. bassiana* (GHA) and *B. thuringiensis* (Bt-k) were sprayed on potatoes to protect against *L. decemlineata*. Identical findings with dual applications of *B. thuringiensis* and *B. bassiana* were also reported in the same host by Furlong and Groden (2003). Other researchers observed similar results when combining *B. thuringiensis* and *B. bassiana* with synthetic insecticides (Fargues, 1975; Lewis and Bing, 1991). Contrarily, no synergism was observed between *B. thuringiensis* and *B. bassiana* against 4th stage larvae of *L. decemlineata* (Costa et al., 2001). In such cases the method of fungal spore application may retard the synergistic effect and cause a differential response in terms of the level of mortality. Different responses of *Chilo partellus* were reported when fungal spores were applied on leaf disk compared to spray and dipping (Tefera and Pringle, 2003).

Developmental parameters of *H. armigera* were greatly affected by the treatments applied and were associated with rate of application. Similar to our findings, Khalique and Ahmad (2002) reported extended larval durations with increased Bt-k concentrations. The studies of Ma et al. (2008) and Marzban et al. (2009) also reported growth retardation of *Ostrinia furnacalis* and *H. armigera* when challenged with Cry1Ac from *B. thuringiensis*-treated diets and combined action of *B. thuringiensis* (Cry1Ac) and HaCPV, respectively. In the larval development assay, our findings indicated toxic effects of the pathogen as indicated by decreased frass production. These findings corroborate those of Marzban et al. (2009) who reported more food uptake in check treatments as compared to the treatments applied. Similarly, reduced frass production in *Trichoplusia ni* were reported with the increase of treatment concentrations (Janmaat et al., 2014). Surprisingly, we observed a peak in frass production in the combined treatment on day 11. It is not clear why this occurred yet it is known that food ingestion and thus frass production can be variable over an insect’s life cycle (Moore et al., 1992; Hernández-Velázquez et al., 2002; Mohammadbeigi and Port, 2015). Nonetheless, overall we observed negative impacts of the treatments on larval fitness. These responses may be attributed to increased *B. thuringiensis* concentrations altering the protein to carbohydrate ratio of the diet, which resultantly disturbs the insect’s growth response (Simpson and Raubenheimer, 1995). Reduced food consumption with *B. thuringiensis* treatments (Nathan et al., 2005; Ramalho et al., 2011), food utilization (Prutz and Dettner, 2005; Ramalho et al., 2011) and reduced larval weight has been reported previously.

This study provides evidence from the laboratory that combining *B. bassiana* and *B. thuringiensis* may provide high levels of synergistic control of *H. armigera*. A unique aspect of the study is that we utilized *B. thuringiensis* in combination with endophytic *B. bassiana* rather than aqueous fungal applications (as has been used in prior studies). Our laboratory findings of efficacy must be extended to field conditions in future research. Also, this novel approach of combining endophytic fungi with other microbial control applications should be investigated in other pest and cropping systems. This paper is fundamental and meant as proof of concept. As far as practical use, other methods for inoculating the plants with *B. bassiana* (such as foliar spray or seed dressing etc.) can be used on a larger scale.

## Data Availability Statement

All datasets generated for this study are included in the article/supplementary material.

## Author Contributions

WW conceived and designed research. MT and WW conducted experiments. MT, DS-I, and WW analyzed data and wrote the manuscript. WW, DS-I and AA-S edited and finalized the manuscript. All authors read and approved the manuscript.

## Conflict of Interest

The authors declare that the research was conducted in the absence of any commercial or financial relationships that could be construed as a potential conflict of interest.
